# A Very Rare Case of an Anomalous Right Coronary Artery Originating From the Proximal Aortic Arch

**DOI:** 10.7759/cureus.75080

**Published:** 2024-12-04

**Authors:** Houssein Y Toufayli, Georges y El Hachem, Emmanuel Salengro

**Affiliations:** 1 Cardiology, Centre Hospitalier Intercommunal de Villeneuve-Saint-Georges, Paris, FRA

**Keywords:** anomalous coronaries, cardiac computed tomography angiography, chest discomfort, congenital anomalies (ca), interventional cardiologist

## Abstract

The anomalous aortic origin of a coronary artery is a rare congenital condition characterized by deviations in the origin, course, or size of coronary arteries. Among these, the anomalous aortic origin of the right coronary artery is a less common variant, particularly when originating from the ascending aorta above the sinotubular junction.

## Introduction

The anomalous aortic origin of a coronary artery (AAOCA) is a congenital defect involving abnormalities in the origin, course, or size of one or more coronary arteries. This rare condition has an estimated prevalence of 0.1% to 0.3% in the general population [1]. Most cases are either asymptomatic or discovered incidentally during coronary imaging. However, AAOCA is associated with an elevated risk of arrhythmias, syncope, and sudden cardiac death (SCD), particularly in young athletes, due to potential hemodynamic compromise from anomalous courses of coronary arteries [2].

Among these anomalies, the anomalous aortic origin of the right coronary artery (AAORCA) is more common than the left-sided variant (AAOLCA), with a reported prevalence of 0.1% to 0.9% [3]. In contrast, AAOLCA is significantly rarer, with prevalence estimates ranging from 0.03% to 0.05% in population studies, depending on diagnostic imaging methods and definitions. Importantly, the left-sided variant is often linked to higher risk profiles, particularly when the coronary artery traverses an interarterial course between the aorta and pulmonary artery, predisposing to ischemia or SCD [4].

The most frequent subtype of AAORCA involves the right coronary artery (RCA) originating from the left coronary cusp. However, the case presented here is unusual in that the RCA originates from the ascending aorta above the sinotubular junction, a location rarely reported in medical literature.

Here, we describe the case of a 63-year-old patient presenting with exertional dyspnea, in whom an anomalous RCA arising from the aortic arch was identified. This report underscores the importance of multimodal imaging and careful assessment in diagnosing and managing rare coronary anomalies.

## Case presentation

We report the case of a 63-year-old male with a history of heavy smoking, hypertension managed with perindopril 4 mg and amlodipine 5 mg daily, and dyslipidemia treated with atorvastatin 20 mg daily. The patient presented with several weeks of exertional dyspnea and occasional fatigue but no chest pain or palpitations. On physical examination, his blood pressure was 122/57 mmHg, with no clinical signs of pulmonary edema or heart failure. Cardiac auscultation revealed no murmurs, while bilateral fine wheezes were noted on lung auscultation. An ECG showed sinus bradycardia at 45 bpm without evidence of ischemic changes.

A chest X-ray excluded pulmonary edema, showing clear lung fields and a normal cardiac silhouette. Transthoracic echocardiography revealed no structural abnormalities, with normal left ventricular ejection fraction and no evidence of valvular disease. Given the persistence of exertional symptoms, the patient underwent a treadmill exercise stress test, which was positive for exercise-induced ischemia based on the development of ST-segment depression during moderate exertion.

**Figure 1:**
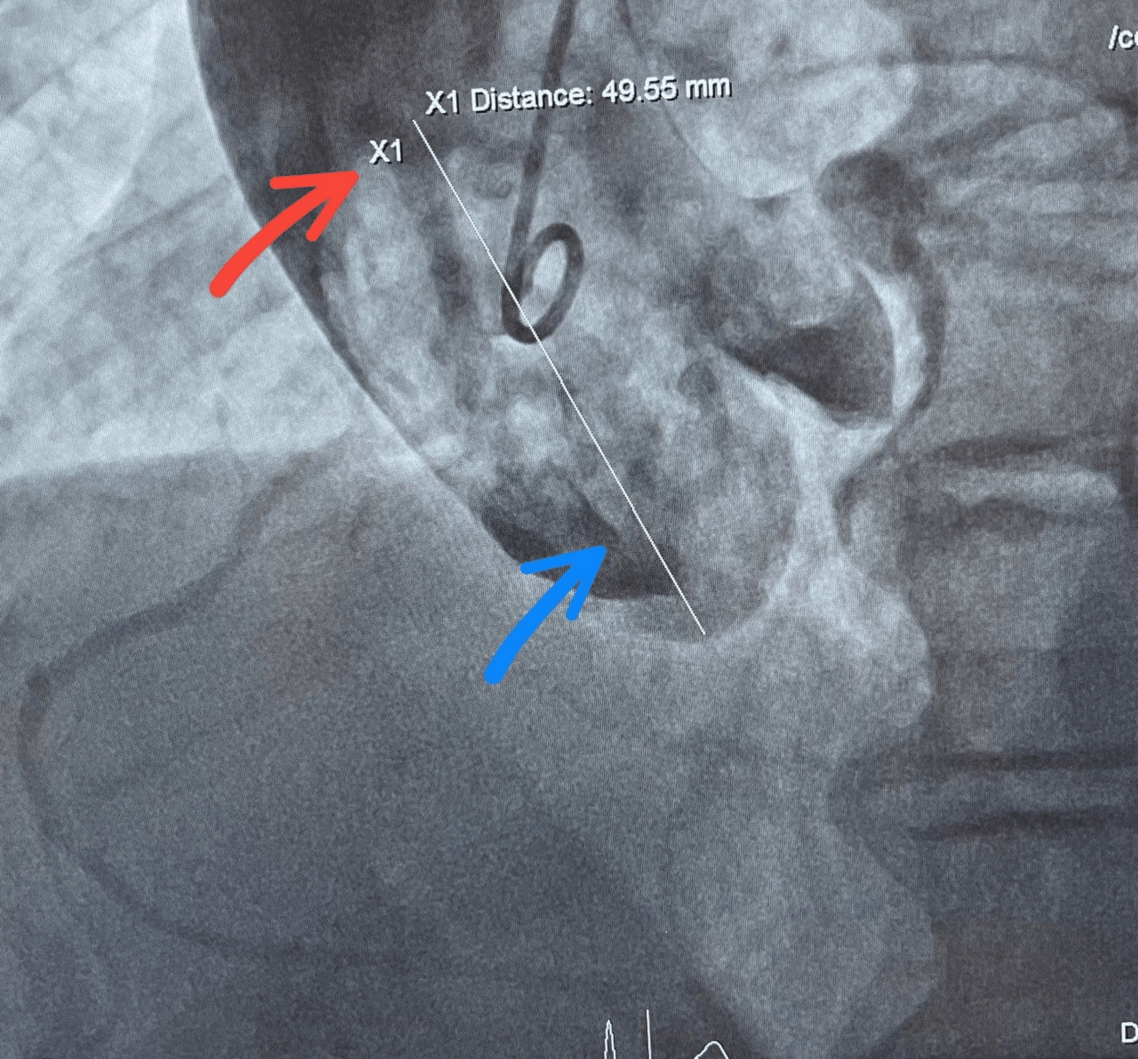
Coronary angiography with intra-aortic contrast injection RCA: right coronary artery Red arrow: ostium of RCA originating from the aortic arch Blue arrow: coronary cusp

**Figure 2:**
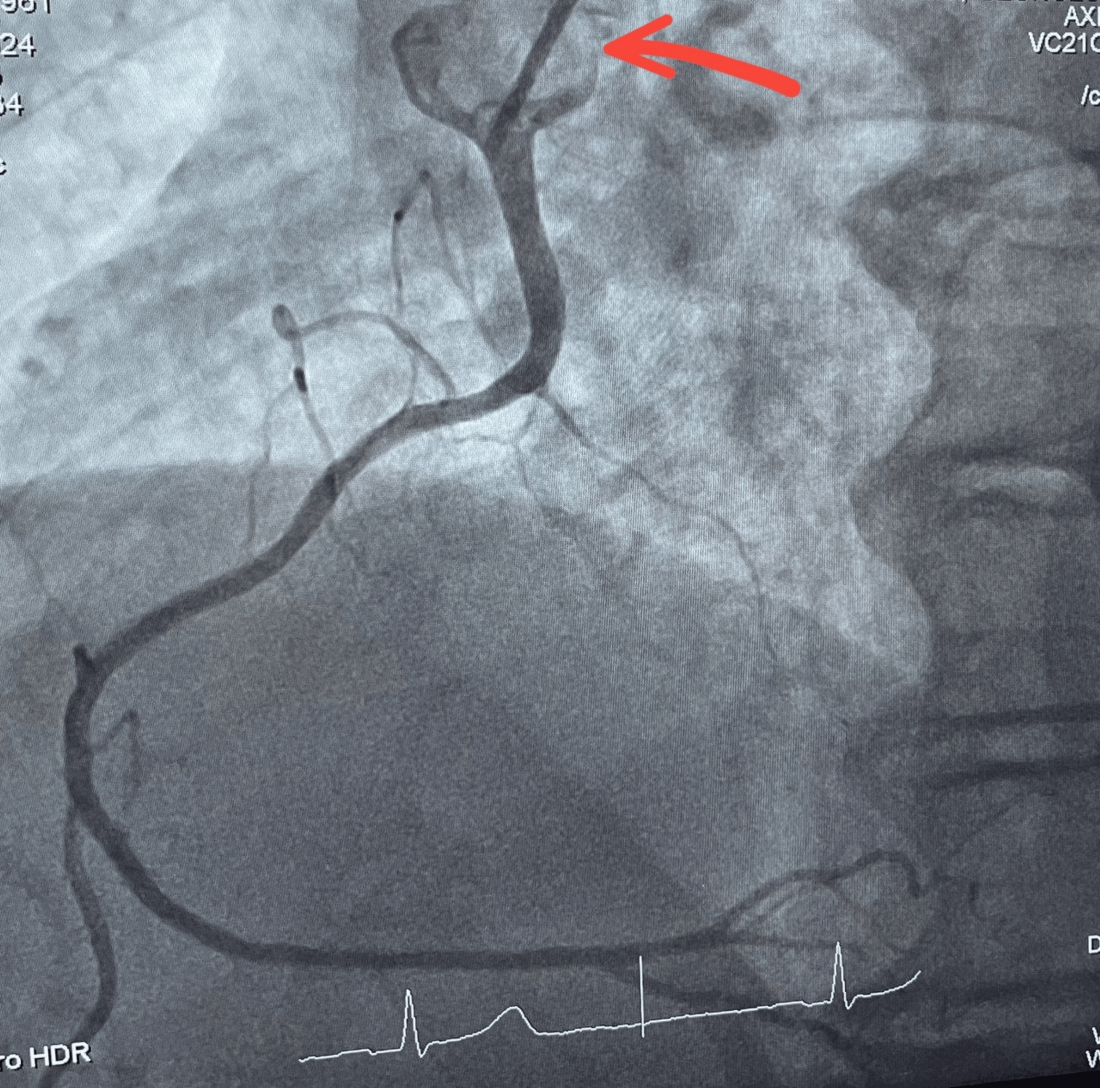
Coronary angiography of the RCA RCA: right coronary artery Red arrow: multipurpose catheter in the RCA

Based on these findings, the patient was referred for diagnostic coronary angiography via right radial access. The angiogram revealed diffusely infiltrated coronary arteries without significant stenosis. A dominant RCA was noted, originating approximately 45 mm above the coronary cusp, as identified through non-selective injection using a pigtail catheter (Figure 1). Selective RCA angiography was performed using a multipurpose catheter, confirming the anomalous high origin (Figure 2).

**Figure 3:**
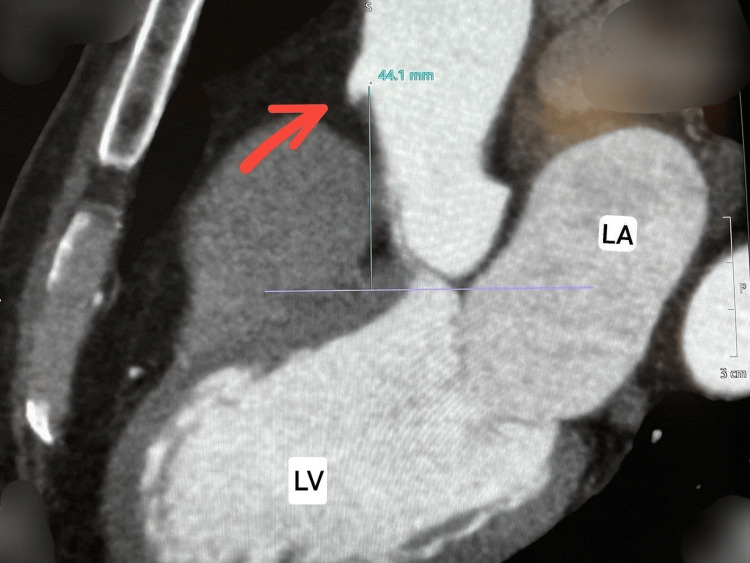
Coronary CT scan image LA: left atrium; LV: left ventricle; RCA: right coronary artery Red arrow: ostial RCA that originates at 44 mm above the aortic cusp

To further characterize the anomaly and its course, the patient underwent coronary computed tomography angiography (CCTA). The CCTA confirmed a very high origin of the RCA at the junction between the ascending aorta and the aortic arch, approximately 44 mm above the coronary sinuses (Figure 3).

**Figure 4:**
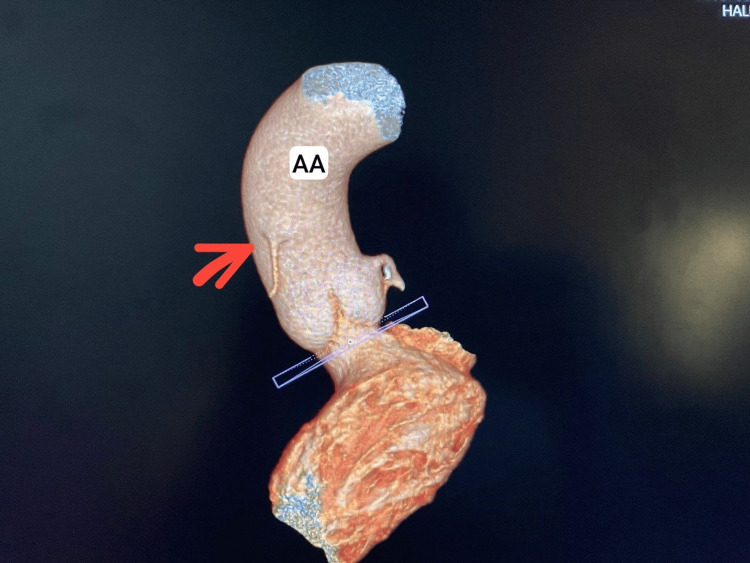
3D reconstruction AA: aortic arch Red arrow: right coronary artery

A 3D reconstruction provided a detailed visualization of the RCA's anomalous course, which followed a benign pattern with no interarterial compression (Figure 4).

The patient’s symptoms were attributed to his underlying hypertension and exertional demand, rather than the anomalous coronary origin, as the anomaly exhibited no high-risk features such as interarterial, retroaortic, or intramural courses. He was managed conservatively with optimization of his antihypertensive and lipid-lowering therapies.

At follow-up three months later, the patient reported significant improvement in his exertional symptoms and no new cardiovascular events.

## Discussion

The AAORCA is often identified either when patients present with symptoms or as an incidental finding during imaging studies. 

When a coronary artery cannot be located within the sinuses of Valsalva during angiography, an anomaly is suspected. Typically, the anomalous orifice is found within the proximal 2 cm of the ascending aorta. In cases of a high ostium, the coronary artery's distribution generally remains normal [5]. 

Among coronary anomalies, those involving the circumflex artery are more common, while RCA anomalies are relatively rare. Studies indicate that the RCA most frequently arises anomalously from the left sinus of Valsalva, with a reported prevalence between 6% and 27%. In some cases, the RCA has been found to originate from the left anterior descending artery or the circumflex artery, accounting for 12.5% of RCA anomalies. However, cases where the RCA originates from the ascending aorta above the sinotubular junction are exceedingly rare [6]. 

In a study by Serkan et al., the prevalence of this anomaly was reported to be 0.0045% (4 cases out of 8,776 patients), representing only 2.1% of all anomalous coronary origins [7]. 

CCTA is particularly useful for detailed visualization of coronary anatomy and its spatial relationships with adjacent structures. It also facilitates 3D reconstructions, which enhance diagnostic precision. Management options for coronary anomalies include surgical techniques such as unroofing, reimplantation, bypass grafting, Takeuchi repair, and interventional or surgical closure of fistulas [8]. 

Recognizing anomalous coronary origins is vital for procedural success. Accurate identification is critical for selecting the appropriate diagnostic or guiding catheter to engage the ostium of the anomalous artery and to minimize complications, particularly in urgent clinical settings. 

## Conclusions

This case highlights a rare presentation of an anomalous RCA originating from the aortic arch, diagnosed in a 63-year-old male with exertional dyspnea. Multimodal imaging, including coronary angiography and computed tomography angiography, was essential for accurately diagnosing and characterizing this anomaly. While the condition is often asymptomatic, its recognition is critical for guiding diagnostic and therapeutic strategies, particularly in symptomatic patients. This report underscores the importance of vigilance and advanced imaging techniques in identifying coronary anomalies to optimize patient outcomes.
